# Deep learning-based approach for differential diagnosis of odontogenic cysts from histopathological images

**DOI:** 10.4317/medoral.27697

**Published:** 2025-11-22

**Authors:** Damla Torul, Ibrahim Sevki Bayrakdar, Mustafa Hakan Bozkurt, Havva Erdem, Muruvvet Akcay-Celik, Busra Ersan-Erdem, Fadime Gul Salman

**Affiliations:** 1 Department of Oral and Maxillofacial Surgery, Ordu University, Ordu, Turkey; 2 Department of Oral and Maxillofacial Radiology, Eskişehir Osmangazi University, Eskişehir, Turkey; 3 Department of Artificial Intelligence and Data Engineering, Karadeniz Teknik University, Trabzon, Turkey; 4 Department of Pathology, Ordu University, Ordu, Turkey; 5 Department of Medical Pathology, Uskudar University, Istanbul, Turkey; 6 Maveria Technologies, Trabzon, Turkey

## Abstract

**Background:**

This study aims to provide Deep Learning (DL) based artificial intelligence (AI) methods using histopathology images to diagnose different types of odontogenic cysts (OCs) differentially.

**Material and Methods:**

Within the scope of the proposed study, hematoxylin and eosin (H&amp;E) stained images of 3 different cyst groups were used. The dataset consists of histopathology images of 87 Dentigerous cysts (DC), 198 radicular cyst (RC), and 63 odontogenic keratocyst (OKC). Each image was zoomed with 3 different zoom levels and resized to 224x224 as preprocessing. In addition to the classical CNN method, Inception V3, VGG16, VGG19, and Xception architectures were used. The data set was split into training, validation, and test groups to avoid retesting.

**Results:**

The average accuracy, precision, sensitivity (recall), and F1-Score values obtained for CNN were 0.77, 0.80, 0.77, 0.75, and for VGG16 were 0.89, 0.90, 0.89, 0.89. For VGG19, these values were determined as 0.89, 0.90, 0.89, and 0.88, for Xception, these values were determined as 0.62, 0.52, 0.62,and 0.52 and for Inception, these values were determined as 0.62, 0.62, 0.62 and 0.56.

**Conclusions:**

It was observed that VGG16 and VGG19 models showed superior performance on the data set in question, while Xception and Inception V3 models converged slower, meaning the training process progressed slower. Results showed that deep neural networks can be efficiently used in detecting OCs. AI-based OC detection may be a decision support tool that reduces interprofessional variability, expedites the diagnostic process, and lessens clinician workload.

## Introduction

Remnants of the tooth development process may result in an odontogenic cyst (OC) which is defined as an epithelium-lined pathological cavity (1 , 2), A radicular cyst (RC) is the most prevalent inflammatory OC lined by non-keratinized stratified squamous epithelium and constitutes nearly 50% of the inflammatory OCs (3 - 5). A dentigerous cyst (DC), which makes up around 20% of all OCs, is a developmental cyst that encircles an unerupted tooth. The histological examination of DC reveals two to three layers of cuboidal to squamoid cells next to fibrous connective tissue, but the inflammatory cyst exhibits a thicker epithelium with hyperplastic meshwork and occasionally hyalinized keratin (6 , 7). Making between 3-11% of all OCs, odontogenic keratocyst (OKC) is a comparatively uncommon type of jaw cyst (8). OKCs have a distinctive microscopic appearance consisting of five to eight layers of keratinized epithelium and a basal layer of elongated columnar/cuboidal cells with polarized nuclei that resemble typical "tombstones" (5).

Proper diagnosis of these pathologies is crucial for prognosis since they exhibit distinct biological characteristics which necessitate different treatment approaches (1). Conventionally, histopathology is the gold standard for verifying the clinical diagnosis. The conventional diagnostic workflow, however, is a laborious process that may encounter difficulties such as diagnosis uncertainty, inter/intraobserver bias, and the need for several expert judgments. Additionally, a significant increase in workload and the possibility of clinician tiredness could compromise the results. Therefore, by offering an additional decision-support mechanism, automating this procedure may lessen the load (5 , 9).

Within the field of computer science known as artificial intelligence (AI), machine learning (ML) techniques are employed to simulate and forecast human intellect. AI is now present in many facets of daily life, including healthcare. AI is more likely to be used in image analysis-based diagnostic fields including pathology, radiology, and the identification of ocular and skin illnesses than in other areas of medical care (10 , 11). A subfield of artificial intelligence ML enables computers to "learn" from historical instances and identify patterns in massive, noisy, or complex data sets that are challenging to discern. Deep learning (DL), a branch of ML, is exceptionally good at evaluating images and is described as the use of multilayer artificial neural networks for a wide range of issues. One of the most often used DL techniques for pattern recognition applications is the convolutional neural network (CNN), which does excellent image analysis (9 , 12). Additionally, one of the AI techniques that has been employed more frequently in recent years is transfer learning (TL). Instead of building a CNN, TL shares "prior knowledge" from the pre-trained model after first training a model on a sizable dataset to gain the classification information (12).

The development of AI technology has opened up new avenues for the translation of laboratory results into clinical diagnosis. Computer-aided diagnosis (CAD) offers an automated second opinion that is useful in identifying and categorizing abnormal alterations (13). Expert shortage and overload can be addressed by the automated instruments, which work in conjunction with clinicians (5). AI-powered solutions can also let specialists work on more complicated cases, which lowers interprofessional variability. Because AI supports clinicians' decision-making, it can therefore be utilized to improve diagnostic efficiency and accuracy (9 , 14). In the present study to differentiate between different types of OCs, we aimed to create a DL model using histopathological images stained with H&amp;E. The main goal of the suggested concept was to offer a CAD system that might act as a local or distant decision support mechanism by offering clinicians more information and insight.

## Material and Methods

This study was approved by the ethics committee of Ordu University (No. 2021/260) and conducted by the Helsinki Declaration.

Material

This study was carried out with a data set created from anonymized histopathological images. H&amp;E-stained histopathological images of OKC, DC, and RC were included in the study. Images with low quality that would hinder evaluation were excluded from the study. In this regard, a data set consisting of 87 DC images from 29 patients, 198 RC images from 66 patients, and 63 OKC images from 21 patients were included. The images were annotated by pathologists and categorized into OKC, DC, and RC. Within the scope of the proposed study, color microscope images of 3 different cyst groups were used. Each image was zoomed with 3 different zoom levels. The data was resized to 224x224 as preprocessing.

Method

In the proposed study, a data set was created with the images of different cyst types determined and classified by pathologists. This target data set was expanded with various data enrichment techniques due to the limited number of images. First, a network model was developed and evaluated using a classical CNN method (15). Also, the TL technique was applied using neural network models such as; Inception V3 (16), VGG16 (17), VGG19 (17), Xception (18), which are known to show better results and are current, were used.

A two-stage approach is applied in the study. First, the performances of the methods are evaluated individually with the K-fold validation approach. K is selected as 5 since we use 80% training and 20% test data. In this test an additional validation set used. K-fold validation tests and tests on training/validation/test were performed to assess consistency. Then, the data is separated into training, testing, and validation sets and a separate training is performed.

As image pre-processing, image resizing is applied on the images in accordance with the model to be applied. Then rotation, width shift, height shift, shear, zoom and flip operations are applied as data augmentation operations. Thus, a better training process was tried to be provided by increasing the number of images. Training, test and validation sets are augmented separately and do not mix with each other. The training process is started with the augmented data.

With the results obtained from these trainings, both a second individual evaluation and comparisons of the methods are made. The images in the data set are first subjected to data separation and then data augmentation. This ensures that training, testing, and validation data are not mixed. Then, classification is performed using DL models. The classification performance of each method is evaluated in detail. Performance comparisons of the classifications performed are analyzed.

Training

In the presented study, a CNN architecture is trained from scratch. In addition, TL is applied using 4 different commonly used neural network architectures. CNN structure and hyperparameters are as follows:

CNN Architecture and Hyperparameters

The Proposed CNN Architecture

- Convolutional layer with 32 filters, kernel size (3x3)

- Max pooling layer with a pool size of (2x2)

- Convolutional layer with 64 filters, kernel size (3x3)

- Max pooling layer with a pool size of (2x2)

- Convolutional layer with 128 filters, kernel size (3x3)

- Max pooling layer with a pool size of (2x2)

- Flatten layer

- Dense (fully connected) layer with 512 units

- Dropout layer with a dropout rate of 0.5

- Dense layer with 256 units

- Dropout layer with a dropout rate of 0.5.

- Dense output layer with 3 units

Hyperparameters:

- Optimizer: Adam Optimizer with 0,0001 learning rate

- Loss Function: Categorical Crossentropy

- Metric: Accuracy

In the TL-orientated study, the given deep neural network architectures are taken as a basis. The default parameters belonging to the structure of each architecture are used. There are some common layers and parameters applied to the basic structure of the training process. These are given as follows:

- Base model with default parameters (Pretrained with ImageNet)

- Additional layer 1: Dropout layer with a dropout rate of 0.

- Additional layer 2: Dense layer with 3 units

Hyperparameters used common to all architectures:

- Optimizer: Adam Optimizer with 0,0001 learning rate

- Loss Function: Categorical Crossentropy

- Metric: Accuracy

Data augmentation is applied to the data for training. The data is first divided into training, test, and validation sets. For K-Fold, is divided into training and tests for each fold. Then, data augmentation is performed separately on each image set. The data is first separated and then the data is dispatched to data augmentation. Thus, it is ensured that there are no common images in the training, test, and validation sets.

The procedures applied for data augmentation are summarised as follows:

- Rotation range: 20

Randomly rotate images within a 20-degree range

- Width shift range: 0.2

Randomly shift images horizontally by up to 20% of their width.

- Height shift range: 0.2

Randomly shift images vertically by up to 20% of their height.

- Shear range: 0.2

Randomly apply shearing transformations (tilting) by up to 20 degrees.

- Zoom_range: 0.2

Randomly zoom in or out on images by up to 20%.

- Horizontal flip: True

Randomly flip images horizontally (mirror them).

Statistical Analysis

The performance of the network structures was examined by the K-Fold Cross-validation method. TP (True Positive), TN (True Negative), FP (False Positive), and FN (False Negative) values were determined for each fold, and accuracy, precision, recall, and f1-score metrics were calculated accordingly. During the K-Fold process, each accuracy and validation loss value was recorded and averaged, and graphs were created. In the tests performed after K-Fold, performance evaluation was made with Confusion Matrix and ROC curve graphs.

## Results

Networks and Training Settings for Dental Cyst Classification

Basic CNN Architecture

The numerical values of the K-fold cross-validation experiment are given in Table 1. These showed that a balanced performance, although not very high, is achieved with the CNN model.

Table 1

**Figure 1:**
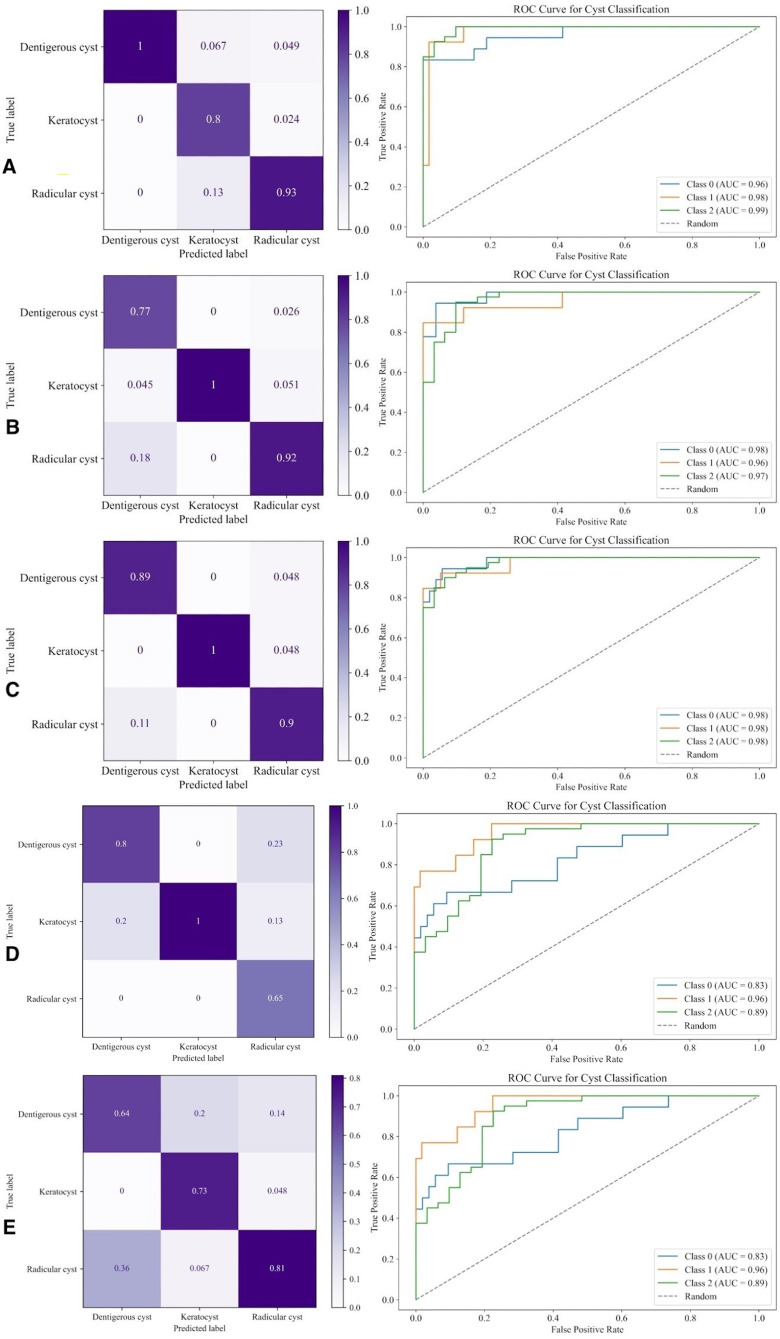
A: Confusion matrix and (CNN) ROC Graph and AUC (CNN). B: Confusion Matrix (VGG16) and ROC Curve and AUC result (VGG16). C: Confusion Matrix (VGG19) and ROC Curve ve AUC result (VGG19). D: Confusion Matrix (Xception) and ROC Curve ve AUC result (Xception). E: Confusion Matrix (Inception V3) and ROC Curve ve AUC result (Inception V3).

With a subsequent test performed after the K-fold validation experiment, the data was divided into 3 groups: Training, validation, and testing, and training was performed again. TP, TN, FP, FN results and precision, recall, and F1-score values obtained from the test data set are given in Table 2. It was observed that precision, recall, and F1-score values were all high for this class (Figure 1A). When Figure 1A was evaluated, it was seen that the performance of the classifier in detecting individual classes was high.

Table 2[caption id="attachment_1766" align="alignnone" width="175"]1 Screenshot[/caption]

Pre-Trained Networks

Apart from CNN, TL has been applied to other network models examined. A trained network model is used for TL. Pre-trained weights were used.

Results for OCs Classification with VGG16

Numerical values of the K-fold cross-validation experiment with the VGG16 model are given in Table 1. These values indicate that the VGG16 model provides a high and balanced performance with its pre-trained structure.

In the second experiment with VGG16, the data was divided into 3 groups: Training, validation, and testing, and training was performed again. With this experiment, performance evaluation on test data was made. TP, TN, FP, FN results and precision, recall, and F1-score values obtained from the test data set are given in Table 2. Visualization of the ratios on the test data on the confusion matrix is given in Figure 1B. The AUC values are given in Figure 1B.

Results for OCs Classification with VGG19

Numerical values of the K-Fold cross-validation experiment with the VGG19 model are given in Table 1. VGG19 model gives a high and balanced performance with its pre-trained structure.

In the second experiment with VGG19, the data was divided into 3 groups: Training, validation, and testing, and training was performed. With this experiment, performance evaluation on test data was made. TP, TN, FP, FN results and precision, recall, and F1-score values obtained from the test data set are given in Table 2. The confusion matrix for the given results is shown in Figure 1C. The AUC values for each of the three classes are shown in Figure 1C.

Results for OCs Classification with Xception

Numerical values of the K-Fold cross-validation experiment with the Xception model are given in Table 1. These values show that lower average values are obtained with the Xception model than with the classical CNN model.

In the next experiment conducted with the Xception model, the data was divided into 3 groups: Training, validation, and testing. The performance evaluations of the 3 classes after this training are given in Table 2. ROC Graph and AUC result on test data are shown in Figure 1D. and Figure 1D.

Results for Dental Cyst Classification with Inception V3

Numerical values of the K-Fold cross-validation experiment with the Inception V3 model are given in Table 1. It is understood that the Inception V3 model provides a high and balanced performance with its pre-trained structure.

In the next experiment, the data was divided into 3 groups: Training, validation, and testing, and applied to training. Performance evaluations of the 3 classes after the training are given in Table 2. ROC Graph and AUC results on test data are given in Figure 1E.

Comparison of the Models

Although the performance obtained with the proposed CNN network model without pre-training was not low in both the K-fold approach and secondary tests, better performance was achieved with pre-trained models (Table 3). According to experimental results, it has been observed that VGG16 and VGG19 models show statistically high and balanced performance with the K-Fold cross-validation method and a similar training process. The results of the Xception model are significantly lower than other models. The performance of the Inception V3 model similar to VGG16 and VGG19, lower than the performance of these two models, but better than Xception.

Table 3

As a result, it has been observed that VGG16 and VGG19 converge faster for the proposed data set in the OCs classification problem and achieve higher performance. Although the Xception and Inception V3 models perform well in certain classes, it is seen that their general performance cannot compete with these two models for this problem.

Model Interpratibility

The methods evaluated in the study were examined in terms of interpretability with the help of heat maps. The heat maps provide details regarding the activity of the AI model on the image. Furthermore, this visualization method reveals which areas receive more attention from the model and which features are influential in the decision-making process (Table 4).

Table 4

When evaluated for Dentigerous cyst:

In examining the heat maps for classical CNN, it is observed that the model's activation spreads across the entire image. This indicates that the model pays equal attention to all parts of the image during its decision-making process. However, this can lead to certain important features being overlooked. In Inception V3, VGG16, and VGG19 models, it is generally observed that attention is focused on areas near outlines. These models try to achieve more effective results by concentrating on specific regions within the image. The Xception model exhibits a different approach altogether, demonstrating a distinct circular attention pattern. Instead of focusing on the center of the image, it concentrates on a circular area surrounding it.

When evaluated for Keratocyst:

The CNN model again shows a wide attention range in its heat map. However, it can be said that there is more focus on central regions close to outlining areas. The Inception V3 model has adopted a more focused approach by giving intense attention to regions near outlines while displaying less attention to other areas. Similarly, both VGG16 and VGG19 models have focused on linear regions and have concentrated more on boundaries compared to Inception V3. For Xception, insufficient activation has been observed.

When evaluated for Radicular cyst:

The CNN model again paid attention to a wide activation area; however, it appears that focus concentrated mainly around boundaries in the middle region for this cyst image. There is less activation observed in areas not close to boundaries. The Inception V3 model shows greater attention to cell borders situated in lower-middle regions of the image. The VGG16 and VGG19 models have also focused specifically on cell cavities within their images for differentiation purposes. Additionally, it has been noted that Xception's activation map prominently focuses on edge areas-similar to what was seen with Dentigerous cyst-concentrating on a circular area of attention as well. These findings indicate that each model adopts different strategies in their decision-making processes.

## Discussion

AI is rapidly expanding in the medical field as a supporting mechanism for decision-making processes, even if it cannot entirely replace the human cognitive system (19). Particularly in fields where decision-making significantly depends on the interpretation of complex visual data, automated tools can assist decision-making and can reveal new perspectives to support precision medicine (20). Furthermore, AI is reported to have the capacity to process more than 250 million images in a day, which can be very helpful in clinical management for extremely challenging diagnostic cases and in places where clinicians are in low supply (20). An AI-based CAD system can also free up clinicians' time by allowing them to focus more on difficult-to-diagnose cases and spend less time reviewing benign tissue, which is typically straightforward to distinguish from malignancy (9).

In the literature, deep learning studies on dental cyst histopathologic images have not yet reached sufficient depth; in particular, the performance of advanced architectures such as Xception, VGG19 and Inception in this field has not been comprehensively evaluated. Studies on OKC, DC and RC detection usually focus on individual cyst types and holistic approaches to classify these three cysts together are limited. Moreover, there is a lack of validation of the performance results obtained in histopathologic image classification. This study aims to classify three common odontogenic cysts (DC, RC, and OKC) in a multi-class manner with computer-aided systems, deeply investigates the effectiveness of TL, makes the decision-making processes of DL models explainable, improves the reliability of performance results with K-fold cross validation method, and increases the generalizability and robustness of the developed method by analyzing histopathological images at three different zoom levels. Therefore, we sought to create an AI pipeline that has demonstrated promising results in the automation of histopathological image classification by eliminating the subjective evaluation of the specimens and interlaboratory variations by standardizing the procedure, on incisional biopsies of H&amp;E stained histopathology images to differentially diagnose the OCs.

A small amount of research on histopathologic image categorization on odontogenic pathologies diagnosis using various AI models has been published in the literature. In the study of Sukegawa et al. (21) CNN-DL models VGG16 and ResNet50 to identify oral squamous cell cancer from histopathological images were used. With an accuracy of 0.8622 and an AUC of 0.9602, they discovered that VGG16 performed the best. They also noted that the clinicians' diagnostic abilities much increased when DL models were employed as supplemental diagnoses. A system to identify both recurrent and non-recurring OKC was attempted to be constructed by Mohantany et al. (8) by using a customized dataset of 48 annotated whole slide images. They employed transformer-based self-attention processes with sequential modeling via long short-term memory (LSTM). The algorithm's results indicated accuracy, recall, and area AUC of 0.98, 1.0, and 0.98, respectively. To automate the detection of recurrent OKC even more quickly, the authors proposed integrating their system into a risk stratification process. The same research group is working on another investigation to create an automated OKC diagnosis system that can be utilized as a decision support tool. The ReliefF feature selection algorithm (ReliefF) in CNN and principal component analysis (PCA) were employed. For the suggested model 97% classification accuracy was reported by the authors (7).

For the recurrence of sporadic OKK on H&amp;E-stained pathological images, Rao et al. (22) employed an ensemble DL-based prediction algorithm integrated with DenseNet-121, Inception-V3, and Inception-Resnet-V3 classifiers on 1660 pathologically annotated digital images of OKC. According to the study's findings, DenseNet-121 predicted recurrent OKCs with 93% accuracy. Additionally, the novel ensemble model demonstrated 97% accuracy, the standard ensemble model demonstrated 95% accuracy, and an AUC of 0.9872 after integration and training. The authors propose integrating their innovative ensemble model into a CAD system to automate OKC risk stratification. In another work, the same group used a pre-trained VGG16, DenseNet-169 model to build an automated system for histopathology image classification that could recognize OKC in H&amp;E-stained 54 OKc, 23 DC, and 20 RC microscopic images. They suggest adding the proposed algorithm to the automation system for OKC and non-OKC diagnostics (5). A total of 2,157 H&amp;E-stained images from 519 cases were gathered by Cai et al. (20) to create digital pathology-based AI models for OKC diagnosis and prognosis. AI models were reported to perform exceptionally well in the diagnosis (AUC=0.935, 95% CI: 0.898-0.973) and prognosis (AUC=0.840, 95% CI: 0.751-0.930) of OKC when the Inception_v3 neural network was used. Frydenlund et al. (1) used AI to differentiate DCs, OKCs, glandular odontogenic cysts, and lateral periodontal cysts from H&amp;E-stained samples. They employed the bagging with logistic regression as a base learner (BLR), and a support vector machine (SVM). Results of their study showed that between 83.8% and 92.3% of the time, the type of cyst was accurately predicted using an SVM, and between 90% and 95.4% of the time, using a BLR, among the four classes of OCs. The authors also observed that the exclusion of DCs from the data set enhanced the classification rate of the algorithms to 96.2% for both SVM and BLR. Sakamoto et al. (23) aimed to develop a digital histopathology system for identifying OKC in H&amp;E-stained tissue specimens of jaw cysts on 5000 microscopy images with 400× magnification from 199 OKCs, 208 DCs, and 55 RCs using VGG16 architecture. They observed 0.997 AUC for the entire algorithm.

In the proposed study, a network model was developed and evaluated using a classical CNN method, and the TL technique was applied to neural network models; Inception V3, VGG16, VGG19, Xception. The average accuracy, precision, sensitivity (recall), and F1-Score values obtained for CNN were 0.77, 0.80, 0.77, 0.75, and for VGG16 were 0.89, 0.90, 0.89, 0.89, respectively. For VGG19, these values were determined as 0.89, 0.90, 0.89, and 0.88. For Xception, these values were determined as 0.62, 0.52, 0,62 and 0.52. For Inception, these values were determined as 0.62, 0.62, 0.62 and 0.56. These results show that the deep structure layers of the VGG family provide performance in OC types. In this study, the performance differences of different artificial intelligence models (CNN, Xception, Inception V3, VGG16, VGG19) in the identification of OCs were examined.CNN model is a basic deep learning approach and is widely used in image classification tasks. In this study, the performance of the CNN model was limited and adequate results could not be obtained despite data enrichment techniques. Xception and Inception V3 models have more complex architectures and resulted in slower training processes. In contrast, the VGG16 and VGG19 models performed better on the dataset. VGG16 and VGG19 models were found to focus more on cell spaces and boundaries in cyst images. As a result, the deep neural network models VGG16 and VGG19 achieved higher accuracy, precision and F1-score values in the detection of OCs. On the other hand, Xception and Inception V3 models showed lower performance for this dataset due to the slower training process.

This study has several limitations. For the generalizability of the model's performance, a multi-center cohort with larger sample sizes should be provided. Additionally, prospective design might enhance the study's findings.

## Conclusions

The VGG16 and VGG19 models performed better than the Xception and Inception V3 models, indicating a slower training rate. The findings demonstrated the high efficiency with which OCs may be detected using deep neural networks. Automatization of this process can reduce the cost of healthcare in resource-constrained areas, cost-effective cloud settings can employ this system. Also, can increase clinicians' confidence in AI-powered diagnostic tools and provide transparency towards clinical practice.

## Figures and Tables

**Table 1:** Table K-Fold cross-validation.

	Fold Number	Accuracy	Precision	Recall	F1-Score	TP	TN	FP	FN
CNN	0	0.71	0.70	0.71	0.67	32	44	7	10
	1	0.78	0.80	0.78	0.78	33	45	4	10
	2	0.78	0.84	0.78	0.76	34	51	8	4
	3	0.82	0.85	0.82	0.80	33	48	5	7
	4	0.78	0.83	0.78	0.75	34	43	6	8
VGG16	0	0.87	0.90	0.87	0.86	37	50	2	5
	1	0.91	0.91	0.91	0.91	38	51	3	3
	2	0.84	0.87	0.84	0.83	36	50	7	5
	3	0.91	0.92	0.91	0.91	36	51	5	4
	4	0.92	0.93	0.93	0.93	43	51	2	4
VGG19	0	0.87	0.89	0.87	0.86	37	50	4	5
	1	0.87	0.89	0.87	0.86	35	49	5	5
	2	0.89	0.90	0.89	0.89	34	51	5	3
	3	0.87	0.89	0.87	0.87	34	49	5	5
	4	0.93	0.94	0.93	0.93	44	51	2	4
Xception	0	0.58	0.58	0.58	0.48	30	43	11	12
	1	0.67	0.58	0.67	0.60	29	43	7	12
	2	0.60	0.55	0.60	0.52	32	49	19	4
	3	0.58	0.44	0.58	0.48	32	45	16	10
	4	0.65	0.44	0.65	0.53	36	44	10	11
Inception V3	0	0.56	0.48	0.56	0.50	30	41	18	11
	1	0.58	0.66	0.58	0.48	31	43	13	12
	2	0.60	0.58	0.60	0.52	30	45	15	10
	3	0.65	0.71	0.65	0.61	32	47	12	8
	4	0.69	0.67	0.69	0.68	36	43	11	8

1

**Table 2:** Table TP, TN, FP, FN ve F1 results.

	Class Number	Precision	Recall	F1-Score	TP	TN	FP	FN
CNN	0 (Dentigerous cyst)	1.00	0.83	0.91	15	68	0	3
	1 (Keratocyst)	0.8	0.92	0.86	12	70	3	1
	2 (Radicular Cyst)	0.93	0.95	0.94	38	69	3	2
VGG16	0 (Dentigerous cyst)	0.77	0,94	0,85	17	70	5	3
	1 (Keratocyst)	1.00	0.77	0.87	10	68	0	3
	2 (Radicular Cyst)	0.92	0.90	0.91	36	67	3	4
VGG19	0 (Dentigerous cyst)	0.88	0.88	0.88	16	69	2	2
	1 (Keratocyst)	1.00	0.85	0.92	11	69	0	2
	2 (Radicular Cyst)	0.90	0.95	0.92	11	69	0	2
Xception	0 (Dentigerous cyst)	0.80	0.22	0.35	4	57	1	14
	1 (Keratocyst)	1.00	0.31	0.47	4	62	0	9
	2 (Radicular Cyst)	0.65	1.00	0.78	40	71	22	0
Inception V3	0 (Dentigerous cyst)	0.64	0.5	0.56	9	62	5	9
	1 (Keratocyst)	0.73	0.85	0.79	11	69	4	2
	2 (Radicular Cyst)	0.81	0.85	0.83	34	65	8	6

2

**Table 3:** Table Comparison of the models.

Model	CNN	VGG16	VGG19	Xception	Inception
Accuracy (K-Fold Mean)	0.77	0.89	0.89	0.62	0.62
Precision (K-Fold Mean)	0.80	0.90	0.90	0.52	0.62
Recall (K-Fold Mean)	0.77	0.89	0.89	0.62	0.62
F1-Score (K-Fold Mean)	0.75	0.89	0.88	0.52	0.56

3

**Table 4:** Table Heatmaps of the models.

Model	Dentigerous Sample	Keratocyst Sample	Radicular Sample
CNN			
Inception V3			
VGG16			
VGG19			
Xception			

4

## Data Availability

Declared none.
